# Molecular Epidemiology, Antimicrobial Susceptibility, and Clinical Features of Methicillin-Resistant *Staphylococcus aureus* Bloodstream Infections over 30 Years in Barcelona, Spain (1990–2019)

**DOI:** 10.3390/microorganisms10122401

**Published:** 2022-12-03

**Authors:** Daniel Antonio Vázquez-Sánchez, Sara Grillo, Anna Carrera-Salinas, Aida González-Díaz, Guillermo Cuervo, Inmaculada Grau, Mariana Camoez, Sara Martí, Dàmaris Berbel, Fe Tubau, Carmen Ardanuy, Miquel Pujol, Jordi Càmara, Mª Ángeles Domínguez

**Affiliations:** 1 Department of Microbiology, Hospital Universitari de Bellvitge-IDIBELL, 08907 Barcelona, Spain; 2 CIBER de Enfermedades Infecciosas (CIBERINFEC), Instituto de Salud Carlos III, 28020 Madrid, Spain; 3 Department of Infectious Diseases, Hospital Universitari de Bellvitge-IDIBELL, 08907 Barcelona, Spain; 4 CIBER de Enfermedades Respiratorias (CIBERES), Instituto de Salud Carlos III, 28020 Madrid, Spain; 5 Department of Medicine, School of Medicine and Health Sciences, University of Barcelona, 08007 Barcelona, Spain; 6 Department of Pathology and Experimental Therapeutics, College of Medicine, University of Barcelona, 08007 Barcelona, Spain

**Keywords:** MRSA, *Staphylococcus aureus*, bacteraemia, antimicrobial resistance, whole-genome sequencing, bloodstream infection, BSI

## Abstract

Methicillin-resistant *Staphylococcus aureus* bloodstream infections (MRSA-BSI) are a significant cause of mortality. We analysed the evolution of the molecular and clinical epidemiology of MRSA-BSI (*n* = 784) in adult patients (Barcelona, 1990–2019). Isolates were tested for antimicrobial susceptibility and genotyped (PFGE), and a selection was sequenced (WGS) to characterise the pangenome and mechanisms underlying antimicrobial resistance. Increases in patient age (60 to 71 years), comorbidities (Charlson’s index > 2, 10% to 94%), community-onset healthcare-associated acquisition (9% to 60%), and 30-day mortality (28% to 36%) were observed during the 1990–1995 and 2014–2019 periods. The proportion of catheter-related BSIs fell from 57% to 20%. Current MRSA-BSIs are caused by CC5-IV and an upward trend of CC8-IV and CC22-IV clones. CC5 and CC8 had the lowest core genome proportions. Antimicrobial resistance rates fell, and only ciprofloxacin, tobramycin, and erythromycin remained high (>50%) due to GyrA/GrlA changes, the presence of aminoglycoside-modifying enzymes (AAC(6′)-Ie-APH(2″)-Ia and ANT(4′)-Ia), and *mph*(C)/*msr*(A) or *erm* (C) genes. Two CC22-IV strains showed daptomycin resistance (MprF substitutions). MRSA-BSI has become healthcare-associated, affecting elderly patients with comorbidities and causing high mortality rates. Clonal replacement with CC5-IV and CC8-IV clones resulted in lower antimicrobial resistance rates. The increased frequency of the successful CC22-IV, associated with daptomycin resistance, should be monitored.

## 1. Introduction

Methicillin-resistant *Staphylococcus aureus* (MRSA) is a serious global health concern that causes considerable morbidity and mortality [1]. MRSA infections are associated with higher mortality than methicillin-susceptible *S. aureus* (MSSA) infections [2,3]. Despite the introduction of new antimicrobials and improved supportive therapy, 30-day mortality in severe MRSA infections, such as bloodstream infections (BSI), remains high, between 18% and 29% [4,5].

*S. aureus* can develop resistance through the acquisition of mobile genetic elements (horizontal gene transfer) or via chromosomal mutations [6,7]. In fact, methicillin resistance is mostly associated with the *mecA* gene, which codes for a low-affinity penicillin-binding protein (PBP2a) incorporated into the chromosome through the acquisition of a genomic island, staphylococcal chromosomal cassette *mec* (SCC*mec*) [8,9]. In addition to β-lactam resistance, a significant proportion of MRSA isolates are also resistant to other antimicrobial groups, such as quinolones, aminoglycosides, and macrolides, a situation that limits the therapeutic options available [10]. In addition, resistance to linezolid, vancomycin, and some last-resort antibiotics for treating MRSA infections, such as ceftaroline or daptomycin, though still uncommon, has already been described [11]. For all these reasons, MRSA has been included in the list of pathogens for which researching and developing new antibiotics is a priority [2,12,13].

MRSA isolates related to the hospital setting frequently exhibit a multidrug-resistant (MDR) phenotype, cause infections in patients with comorbidities, and may be associated with nosocomial outbreaks [14]. The emergence of these MDR isolates seems to be closely related to the selective pressure exerted by the use of antibiotics [15,16]. In contrast, community-acquired (CA) infections may occur in otherwise healthy individuals and are usually less associated with antimicrobial resistance. However, invasive infections caused by CA-MRSA isolates have been reported to be more severe and to require intensive care unit admission more frequently, indicating a potential increased virulence [17,18]. In recent years, broad-scale infection prevention measures have focused on reducing the incidence of infections caused by both hospital-onset (HO-HCA) and community-onset healthcare-associated (CO-HCA) MRSA, thus potentially limiting the spread of clones typically associated with this setting [19]. In this regard, severe MRSA infections are caused by a limited number of clones distributed worldwide [20], whose epidemiology has been established by classical typing methods, such as pulsed-field gel electrophoresis (PFGE), *S. aureus* protein A (*spa*) typing, and multilocus sequence typing (MLST). As with other pathogens, whole-genome sequencing (WGS) has shown excellent discriminatory power for MRSA isolates, and it is increasingly being used as part of infection control practices [21,22].

In this study, we explored the molecular epidemiology, genomic diversity, and clinical characteristics of MRSA-BSI in a tertiary care hospital over 30 years (1990–2019). The use of microbiologic and genomics approaches together with WGS and clinical data allowed us to trace the rise and fall of MRSA clones and to study the mechanisms underlying antimicrobial resistance and the changes occurring in the clinical presentation of MRSA-BSI over time.

## 2. Materials and Methods

### 2.1. Study Setting, Definitions, and Clinical Data

Bellvitge University Hospital (HUB) is a 700-bed teaching hospital for adult patients located in the Barcelona Metropolitan Area in Spain. At HUB, all MRSA-BSI isolates are prospectively collected and stored at −80 °C. MRSA-BSI patients’ demographic and clinical data, including age, sex, acquisition, source of infection, and 30-day mortality, are prospectively collected from electronic resources. For this study, we included episodes recorded over a 30-year period (1990–2019). An MRSA-BSI episode was defined as a positive blood culture for MRSA in a patient with clinical signs and symptoms compatible with infection. Only the first episode per patient and year was considered. BSI episodes were classified as HO-HCA, CO-HCA, or CA, according to Friedman’s criteria and following the new terminology for MRSA bacteraemia [23,24]. In brief, episodes that occurred at least ≥48 h after hospitalisation with no signs or symptoms of infection at admission were considered HO-HCA. Episodes within 48h of admission were considered CO-HCA or CA depending on whether the patient had had recent contact with health-care settings or not, respectively.

### 2.2. Bacterial Isolates and Antibiotic Susceptibility Testing

As part of the daily practice, *S. aureus* isolates were identified by conventional methods (Mannitol agar, DNase and coagulase test) and, since 2012, by matrix-assisted laser desorption/ionisation time-of-flight (MALDI-TOF). Antimicrobial susceptibility testing was performed by disk diffusion (1990–1998) and/or microdilution (from 1999 onwards) using commercial panels (MicroScan, Beckman Coulter, Brea, CA, USA) and following the recommendations and criteria of the Clinical and Laboratory Standards Institute (CLSI) for each period [25]. Susceptibility to 14 antimicrobials (ciprofloxacin, clindamycin, co-trimoxazole, erythromycin, fosfomycin, fusidic acid, gentamicin, linezolid, oxacillin, penicillin, rifampicin, tetracycline, tobramycin, and vancomycin) was routinely tested in all isolates. Susceptibility to ceftaroline, daptomycin, and mupirocin was additionally tested by microdilution and chloramphenicol by disk diffusion in all isolates selected for WGS. Isolates were classified as MDR if they were resistant to ≥3 antimicrobial categories [26].

### 2.3. Molecular Typing, Whole-Genome Sequencing, and Bioinformatic Analysis

Molecular characterisation was performed by PFGE (SmaI) in all available isolates (*n* = 702) (Figure S1). The PFGE band patterns were interpreted according to Tenover’s criteria [27]. Furthermore, other additional tests (MLST, *spa*, *agr*, and SCC*mec* typing) were performed in at least one strain of each PFGE pattern (328/702, 46%) in accordance with a previously described methodology [28]. A selection of 114 isolates (Table S1), representative of all periods and including all major clonal complexes (CC) and sporadic PFGE types, was studied by WGS. DNA extraction and quantification were performed using a QIAmp DNA Mini Kit (Qiagen, Hilden, Germany) and a Quantus™ Fluorometer (Promega, WI, USA) respectively. A Nextera XT DNA Prep Kit (Illumina, San Diego, CA, USA) was used to prepare the libraries, followed by paired-end sequencing on a MiSeq platform (Illumina). Sequences were assembled with the INNUca v4.2 pipeline (github.com/B-UMMI/INNUca; accessed on 7 July 2021) with default parameters and annotated using Prokka v.1.12 [29].

MLST, *spa*-type, and SCC*mec*-type were obtained in silico using online tools from the Center for Genomic Epidemiology [30,31,32]. New alleles and ST profile numbers were registered in PubMLST (pubmlst.org; accessed on 18 February 2022). For phylogenetic analysis, a full genome alignment was constructed using Snippy 3.1 (github.com/tseemann/snippy; accessed on 10 March 2022) and *S. aureus* NCTC8325 (Accession number CP000253.1) as a reference. Subsequently, the whole genome alignment was subjected to the prediction and removal of recombinant regions using Gubbins v2.3.4 software, and a phylogenetic tree was constructed using RAxML-NG [33]. Phylogenetic tree visualisation was performed using Microreact [34]. The pangenome analysis was performed using Roary [35], with a minimum identity percentage of 80% for BLASTp. Genes associated with transferable resistance were screened using ResFinder [36]. The analysis of point mutations in genes associated with antibiotic resistance (*mecA*, *mprF*, *gyrA*, *gyrB*, *grlA*, *grlB*, *pbp2*, *pbp4*, *rpoB*, *rpoC*, *murA*, *glpT*, *uhpT*, *dfrB*, and *ileS*) was performed with Geneious R9 (Biomatters) using *S. aureus* COL (Accession number CP000046) genome as a reference.

Reads were deposited in the European Nucleotide Archive (ENA): study accession number PRJEB54086.

### 2.4. Statistical Analysis

Statistical analyses were conducted using GraphPad Prism 5.01 software. Fisher’s exact test (two-tailed) or an unpaired *t*-test was used when appropriate and *p*-values ≤ 0.05 were considered statistically significant. The incidence rates of MRSA- and MSSA-BSI were calculated using the total number of hospital stays (100,000 patient-days) as the denominator. For the analysis, the study was divided into five periods: 1990–1995, 1996–2001, 2002–2007, 2008–2013, and 2014–2019.

### 2.5. Ethics

This study was performed in accordance with the Declaration of Helsinki of the World Medical Association. It was approved by the HUB’s Clinical Research Ethics Committee (PR293/22). Written informed consent was not required as this was a retrospective and observational study with isolates obtained as part of routine microbiological tests. Patient data were always protected in accordance with the local committee and national standards.

## 3. Results

### 3.1. Incidence and Clinical Characteristics of MRSA-BSI Episodes

**Figure 1:**
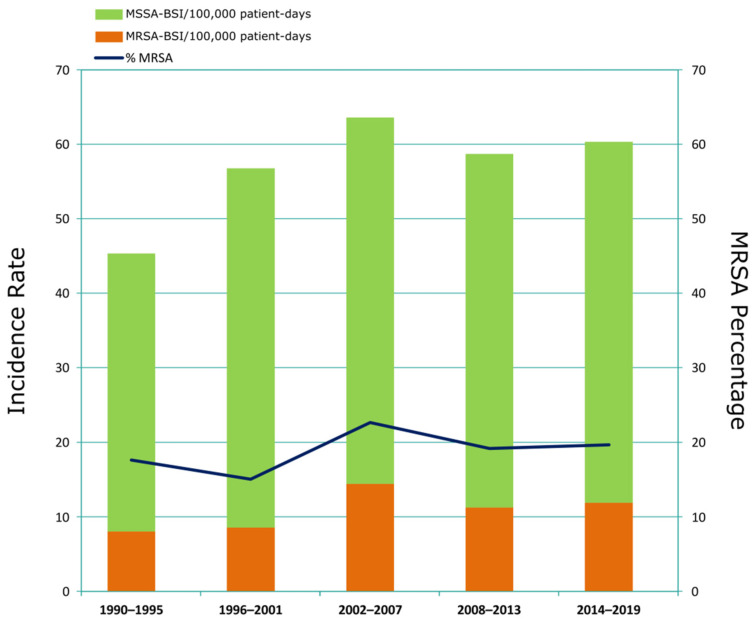
**Evolution of MSSA- and MRSA-BSI over the study period.** Incidence rates of MSSA- and MRSA-BSI (episodes per 100,000 patient-days) are shown in bars. The blue line shows the percentage of methicillin-resistant isolates.

From 1990 to 2019, we detected 3544 MSSA- and 819 MRSA-BSI episodes (Figure 1). The incidence of *S. aureus* BSI increased from 45.3 episodes per 100,000 patient-days in the first period (1990–1995) to 60.3 in the last period (2014–2019). The incidence of both MSSA- and MRSA-BSI increased over time, reaching a peak in 2002–2007 (Figure 1). The percentage of MRSA among all *S. aureus*-BSIs varied between 15% and 23%, with the highest percentage in 2002–2007, coinciding with a peak of *S. aureus*-BSI incidence (63.5 episodes per 100,000 patient-days).

Clinical characteristics were available for 784 MRSA-BSI episodes (Table 1). The median age of patients rose from 60 to 71 years old, and the proportion of female patients increased from 25% to 46% throughout the study period. The proportion of patients with a Charlson’s comorbidity index (CCI) > 2 rose from 10% in 1990–1995 to 94% in 2014–2019. An increase in 30-day mortality was observed after 2008, with rates of 28–29% recorded from 1990 to 2001 and 36–38% between 2008 and 2019. Regarding the acquisition of MRSA infection, the proportion of HO-HCA MRSA-BSI episodes fell from 91% to 26%, whereas the CO-HCA MRSA-BSI episodes rose from 9% to 60% in the same periods. CA non-healthcare-associated episodes emerged in 2002–2007 and increased progressively, accounting for 14% of MRSA-BSIs in the last period (2014–2019). As for the sources of infection, a significant reduction in the proportion of catheter-related BSIs was observed over time (from 57% to 20%). In the last period, HO-HCA MRSA-BSI episodes most frequently originated in the respiratory tract (13/39) or vascular catheter (11/39), while CO-HCA MRSA-BSI sources of infection were mainly vascular catheter (19/89) and skin and soft tissue infections (SSTI) (17/89). The most frequent sources of CA episodes were SSTI (9/21) and unknown focus (4/21) (Table S2).

### 3.2. Molecular Epidemiology of MRSA-BSI

**Figure 2:**
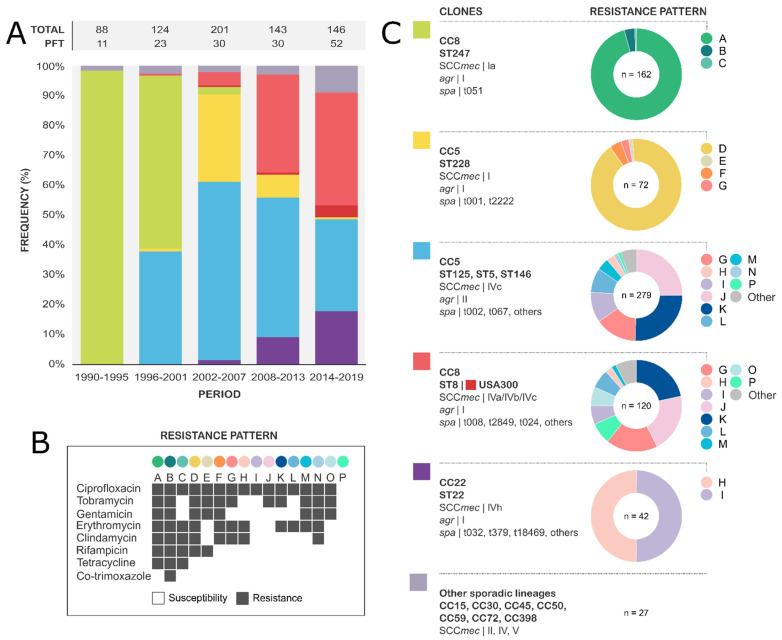
**Evolution of MRSA-BSI clones and antimicrobial resistance patterns.** (**A**) **Distribution and frequency of MRSA-BSI lineages by period.** The total number of MRSA-BSI isolates and the different PFGE types in each period are shown above bars. Clones are represented by coloured bars in (**A**) and by coloured squares in (**C**). (**B**) **Resistance profiles of MRSA-BSI strains.** (**C**) **Molecular characterisation of major clones and their resistance profiles.** The resistance profile of the major clones is represented by coloured dots in (**B**,**C**). Other minor resistance profiles (*n* < 5) are shown as ‘Other’. CC, clonal complex; PFT, pulsed-field type; SCC*mec*, staphylococcal chromosomal cassette *mec*; *spa*, *spa*-type; ST, sequence type; *agr*, accessory gene regulator Agr.

A total of 702/784 isolates (90%) were available for molecular studies. Five main genotypes accounted for 96% (675/702) of the MRSA population (Figure 2). Isolates of Iberian clone CC8 ST247 carrying a SCC*mec* element type Ia (162/702, ST247-Ia [37]) were predominant until 1999 and associated with an MDR profile, including resistance to aminoglycosides, fluoroquinolones, lincosamides, macrolides, rifampicin, and tetracyclines. CC5 ST228-I (72/702, Southern German clone) was prevalent from 2002 to 2009 and also exhibited an MDR profile (macrolides, aminoglycosides, fluoroquinolones, and rifampicin). CC5 isolates harbouring SCC*mec*-IV (279/702, Paediatric clone) emerged in 1997 and increased rapidly to become the most frequent clone from 2001 to 2015. Isolates of this clone showed different STs (mostly ST125 and ST146) and varied resistance profiles (RPs). The second most frequent lineage was CC8 ST8-IV (120/702), which emerged in 2000 and is currently the first cause of MRSA-BSI. Similar to CC5-IV, this lineage includes isolates with diverse antimicrobial RPs, most of them showing tobramycin and ciprofloxacin resistance. In addition, a subset of ST8-IV isolates (18/120) was positive for Panton-Valentine leukocidin (PVL). Six of these isolates were related to the USA300 North American Epidemic clone, which is related to the arginine catabolic mobile element (ACME) and SCC*mec*-IVa, and eight isolates were related to the USA300 South American Epidemic clone, which is associated with copper and mercury resistance element (COMER) and SCC*mec*-IVc. The most recent major clone, CC22 ST22-IVh (42/702, UK-EMRSA-15), has become more frequent since its emergence in 2007, and it is uniformly associated with ciprofloxacin resistance and variable macrolide phenotypes.

Figure S2 shows the pangenome composition of each clone among the sequenced isolates. Pangenome analysis detected a pool of 3778 genes, many shared by all genomes (core genes, *n* = 1679, 44%), 322 genes in 95–99% of the genomes (soft-core genes), 821 genes in 15–94% (shell genes), and 956 genes in <15% of the genomes (cloud genes). The phylogenetic tree shows four major CCs clustering together: CC5, CC8, CC72, and CC22. When each CC was examined separately, differences in the gene pools were observed, with CC72 showing the largest gene pool (*n* = 3255)—although it only contained four samples—and CC22, the smallest (*n* = 2617). CC8 and CC5 had the lowest total core genome proportions (67% and 69% respectively), showing a higher genetic variability than the other CCs, while CC22 was genetically more homogeneous. CC8-ST8 and CC5-ST125 Paediatric clone, had more accessory genes per genome than ST247 or ST228.

### 3.3. Evolution and Molecular Basis of Antimicrobial Resistance

**Figure 3:**
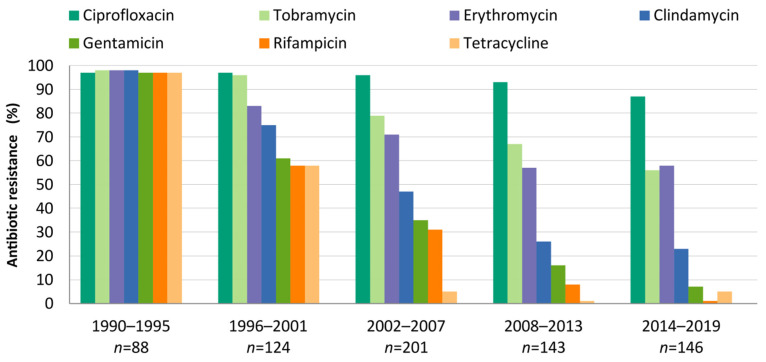
**Evolution of antimicrobial resistance rates by period**. Only antimicrobials with resistance rates above 5% are shown. All decreases in the percentages of antibiotic resistance were statistically significant (*p* < 0.01) when comparing the first (1990–1995) and the last periods (2014–2019).

The evolutions of the antimicrobial resistance rates over time are shown in Figure 3. The percentages of resistance to specific antibiotics among the MRSA isolates are shown by period. The disappearance of genotypes with extended resistance profiles, such as ST247-Ia and ST228I, resulted in a fall in the resistance rates to most antimicrobials. Only ciprofloxacin, tobramycin, and erythromycin had resistance percentages above 50% in the last period. All isolates were susceptible to vancomycin and linezolid.

**Figure 4:**
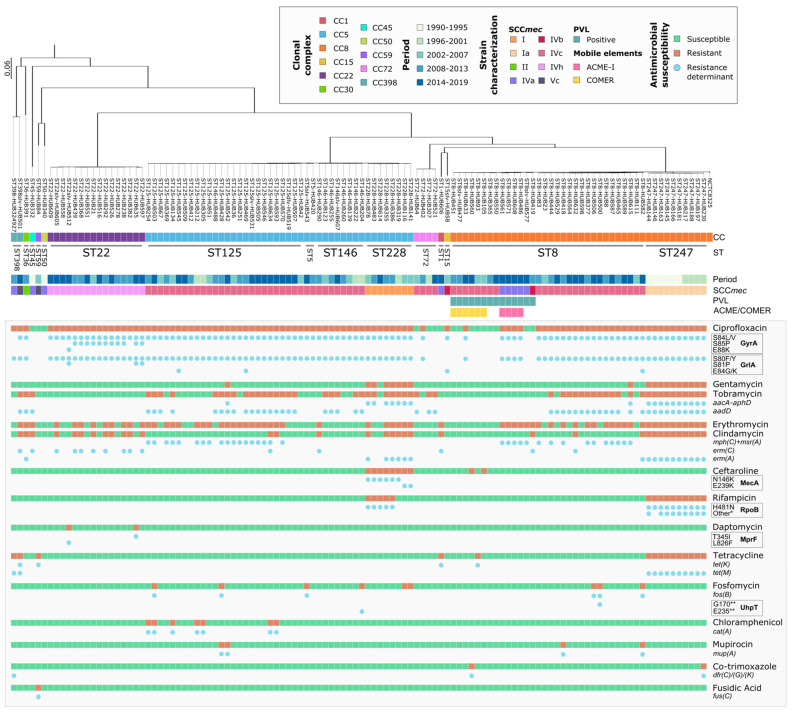
**Assembly-based core SNP phylogenetic tree, period, clonal complex, antibiotic resistance, and associated molecular mechanisms.** The coloured bars indicate the period and the strain characteristics (CC, SCC*mec*, PVL, and ACME/COMER elements). In the grey-shaded area, the coloured squares represent the antimicrobial resistance (in red) and susceptibility (in green) by antibiotic group and following CLSI breakpoints [25], followed by blue circles when a resistance determinant was detected. * Other alterations of RpoB, D471G/Y, and S529L. ** Indicates a STOP codon. CC, clonal complex; ST, sequence type; SCC*mec*, staphylococcal chromosomal cassette *mec*; PVL, Panton-Valentine leukocidin; ACME, arginine catabolic mobile element; COMER, copper and mercury resistance mobile element.

Figure 4 summarises the genetic relatedness and the main resistance determinants of the MRSA-BSI strains sequenced through WGS. The most notable resistance mechanisms among the antibiotics with the highest resistance rates are specified next. **Quinolones**. High-ciprofloxacin MICs (MIC > 2 mg/L) were associated with substitutions in the quinolone resistance-determining regions (QRDRs) of DNA gyrase GyrA (S84L/V, S85P, and E88K) and topoisomerase IV GrlA (S80F/Y, S81P, and E84K/G). One CC398 strain had a first-step mutation in GrlA-S80F (MIC = 2 mg/L). No relevant amino acid substitution in GyrB or GrlB was found. **Aminoglycosides**. Resistance to aminoglycosides was found in all clones except CC22. It was related to the presence of aminoglycoside-modifying enzymes, such as the bifunctional protein AAC(6′)-Ie-APH(2″)-Ia (gentamicin and tobramycin resistance) and ANT(4′)-Ia (tobramycin resistance). **Macrolides and lincosamides**. The *mph*(C) and *msr*(A) genes were found in 32/67 erythromycin-resistant strains. Erythromycin plus clindamycin resistance (MLSB phenotype) was observed in 35 isolates, associated with genes *erm*(A) (mostly detected in ST247-Ia and ST228-I clones) and *erm*(C) (mostly detected in CC22). A single ST8 isolate, in addition to *erm*(A), also carried the *linA* gene. **Rifampicin**. Rifampicin resistance was associated with RpoB alterations, present only in clones ST247-Ia (H481N plus S529L and/or D471G) and ST228-I (H481N). No changes were found in RpoC. **Tetracyclines**. Among the 15 tetracycline-resistant strains, 11 carried *tet*(M), three *tet*(K), and one (CC398) harboured both genes. **Chloramphenicol**. Seven ST125 strains were resistant to chloramphenicol and carried the *catA* gene. **Fosfomycin**. Two fosfomycin-resistant strains had alterations in UhpT (E235STOP or G170STOP) and six carried the *fosB* gene. There were no strains with MurA or GlpT changes. In two ST228 isolates, no genetic determinants were found associated with fosfomycin resistance. **Mupirocin**. Four strains showed high-level mupirocin resistance and the presence of the *mupA* gene. **Co-trimoxazole**. Two resistant strains carried *dfrC* and *dfrG* genes. No mutations were found in the *dfrB* gene. Another CC398 strain had a *dfrK* gene related to an ST398 plasmid [38] but was susceptible to co-trimoxazole. **Fusidic acid**. One strain was resistant and carried the *fusC* gene. **Ceftaroline**. Resistance to ceftaroline was associated with changes in PBP2a (N146K or E239K) in eight ST228-I strains. These isolates were obtained before ceftaroline was implemented at the HUB. Two ST8-IV strains also showed ceftaroline non-susceptibility, but no changes in PBP2a, PBP2, or PBP4 were found. **Daptomycin**. Changes in MprF (T345I and L826F) were found in two daptomycin-resistant CC22-IV strains, isolated during specific treatment with daptomycin.

### 3.4. Major Lineages and Clinical Characteristics of Recent MRSA-BSI Episodes (2008–2019)

We compared the demographic and clinical data of BSI episodes caused by isolates of the most prevalent lineages (CC5-IV, CC8-IV, and CC22-IV) from 2008 to 2019, when these genotypes co-existed (Table S3). In general, episodes caused by all three clones were similar with regard to demographic and clinical characteristics. In addition, no statistically significant differences in age, sex, CCI, or 30-day mortality between the clones were found. Nevertheless, BSI episodes caused by CC8-IV and CC22-IV clones tended to have a lower respiratory tract origin more often than those caused by CC5-IV (frequencies of 17%, 21%, and 9% respectively).

## 4. Discussion

Even though the percentage of methicillin resistance appears to be declining in European countries [39], MRSA-BSI still has very high mortality rates [39,40,41]. Therefore, to understand the changing landscape of MRSA infections, it is essential to study the changes occurring in the profile of patients affected together with the clonal evolution of MRSA isolates. In this study, we present data on the clinical characteristics and the molecular epidemiology of MRSA-BSI at a tertiary-care hospital over the last 30 years.

Following the initial emergence and rise in MRSA-BSI infections during the 1990s [37], methicillin resistance among *S. aureus* clinical isolates has remained stable at around 20% at HUB [5]. Nevertheless, the clinical presentation of MRSA-BSI has changed dramatically. This may be due to changes in the patients, who are currently older and have more comorbidities, to shifts in MRSA clones, or to a combination of the two. In our setting, current MRSA-BSIs are mostly related to health-care acquisition. This is consistent with published data from around the world and might be attributed to population ageing and the increasing use of prosthetic devices, which has also been associated with a rise in the rate of endocarditis [40,41] (as we observed in the 2014–2019 period). Our study also shows a significant reduction in the frequency of catheter-related BSIs over time, a situation that highlights the efficacy of the infection control measures that were progressively implemented in our institution [42]. Indeed, while the incidence of MSSA-BSI has remained stable since 2002, MRSA-BSI incidence has fallen, despite the increasing complexity of patient management. The drastic reduction in catheter-related BSIs together with the population ageing and the increased number of comorbidities (which both raise the risk of complicated bacteraemia) [4,43] may also have influenced the proportional increase in 30-day mortality observed since 2008. Although bacterial factors cannot be overlooked, in our series, we did not find significant differences in clinical characteristics and mortality among infections caused by different MRSA clones, suggesting that host factors mainly drive the observed changes in mortality.

Through the study period, we observed a reduction in the resistance rates for most antimicrobials. This is due to the disappearance of genotypes with extended resistance profiles and large SCC*mec*, such as the ST247-Ia Iberian clone, which caused a significant outbreak in our centre from 1990 to 1995 [37], and the ST228-I Southern German clone, highly prevalent in the 2002–2007 period [44]. Currently, resistance to only quinolones, tobramycin, and erythromycin remains significantly prevalent in MRSA-BSI isolates. Quinolone resistance is widespread among MRSA clones and typically associated with hospital-acquired MRSA [45]. It has been proposed that fluoroquinolone resistance may provide an evolutionary advantage that contributes to the initial phase of the spread of CC5-IV Paediatric or CC22-IV EMRSA-15 clones across European hospital environments [46,47,48]. In regard to tobramycin, its consistency over time and across different clones may originate from the transfer via the conjugation of aminoglycoside resistance determinants between coagulase-negative staphylococci and *S. aureus,* which was demonstrated in another study [49]. In addition, certain SCC*mec* elements, such as type I, comprise additional genes that encode resistance to other antimicrobials, such as *aadD*, and to heavy metal ions [45]. Clones carrying SCC*mec*-IVc can also harbour aminoglycoside-resistant genes in the chromosome or in conjugative plasmids [50]. This may have contributed to the persistence of these determinants in HCA-MRSA clones. With respect to macrolides, the maintenance of erythromycin resistance could be associated with a change in macrolide prescription: in recent years, the consumption of long-acting macrolides (e.g., azithromycin) rose in parallel to a fall in the use of short-acting macrolides [51]. Then, the long-term prophylactic use of azithromycin may have resulted in a selection pressure of macrolide-resistant MRSA in patients with chronic respiratory diseases [52]. Resistance to first-election antibiotics, such as linezolid and vancomycin, and last-resort antibiotics for MRSA-BSI treatment, such as daptomycin or ceftaroline, was negligible in our series. Daptomycin resistance was related to previously described *mprF* mutations [53] in two CC22 strains, and most ceftaroline non-susceptible isolates belonged to a single ancient clone (ST228-I, PBP2A alterations), indicating that these resistance mechanisms are not currently widespread in our area. Nevertheless, it should be noted that an association between *mprF* mutations and CC22 has been reported [54], so an increasing frequency of CC22 strains in our region may affect future daptomycin resistance rates.

A few successful clones were responsible for the majority of BSIs over the study period. Consistent with the published data [39,41], most MRSA isolates in the last decade belonged to CC5-IV or CC8-IV. The competitive fitness advantage of carrying small SCC*mec* elements, such as SCC*mec*-IV [55], together with changes in antimicrobial consumption or infection control strategies [47,48,56], may explain the dominance of CC5-IV and CC8-IV clones over the ancient ST247-Ia. Both clones also showed higher genomic diversity, as evidenced by their high proportions of accessory genomes, which may explain their adaptability. Another reason for the persistence of these epidemic clones in the hospital setting could be their enhanced ability for biofilm production when compared to sporadic MRSA clones. This was shown in a recent paper, where CC5 and CC8 presented the virulence factor *sasG* variant associated with bacteraemia and higher rates of biofilm formation, favouring their success in the hospital setting [57]. In recent years, CC22-IV (EMRSA-15) has emerged as an increasingly important cause of MRSA-BSI in our geographical area. MRSA CC22-IV has been detected worldwide [45], is associated with both healthcare and community acquisition [58] and is one of the main causes of MRSA-BSI in some regions [59]. It has been proposed that the initial expansion of this clone was related to the acquisition of fluoroquinolone resistance, coinciding with the introduction of fluoroquinolones in the hospital setting [46]. However, as most epidemic HCA-MRSA clones also characteristically display fluoroquinolone resistance, it seems that other genomic features must be involved in their success. Nevertheless, the increased incidence of CC22-IV in our geographical area in recent years should be closely monitored.

This study included a large collection of well-characterised MRSA-BSI isolates over a long period of time. However, certain limitations of the study are worth discussing. First, it was restricted by its retrospective nature and by the lack of information on certain MRSA-BSI episodes. Secondly, it did not include episodes occurring outside the hospital, which may represent a bias, but this limitation is probably minor given that the severity of MRSA-BSI usually requires hospital care.

In conclusion, MRSA-BSI has primarily become a community-onset healthcare-associated infection in our setting, affecting the elderly and patients with comorbidities, and leading to high mortality. Antimicrobial resistance rates have fallen, with only ciprofloxacin, tobramycin, and erythromycin continuing to be significant. The emergence and rise of CC22 strains, and their potential link with daptomycin resistance, should be closely monitored.

## Figures and Tables

**Table 1:** **Clinical and demographic characteristics of MRSA-BSI episodes by period.** The table shows the total number of episodes and the percentages (in parentheses).

		Period				
		1990–1995	1996–2001	2002–2007	2008–2013	2014–2019
**Number of episodes**		**137**	**142**	**207**	**149**	**149**
**Sex, female**		34 (25)	38 (27)	76 (37)	62 (42)	68 (46) *
**Age, mean ± SD (range)**		60.2 ± 18.9 (22–94)	68.3 ± 16.3 (23–99)	70.2 ± 14.9 (24–98)	70.1 ± 14.1 (27–94)	71.2 ± 14.5 (19–85) *
**Acquisition**	HO-HCA	124 (91)	113 (80)	98 (47)	70 (47)	39 (26) *
	CO-HCA	13 (9)	29 (20)	107 (52)	71 (48)	89 (60) *
	CA	0 (0)	0 (0)	2 (1)	8 (5)	21 (14) *
**Source of infection**						
Catheter		78 (57)	71 (50)	62 (30)	35 (24)	30 (20) *
Skin and soft tissue		8 (6)	12 (8)	34 (16)	20 (13)	29 (20) *
Respiratory tract		1 (1)	14 (10)	17 (8)	17 (11)	24 (16) *
Urinary		2 (1)	5 (4)	22 (11)	21 (14)	12 (8) *
Osteoarticular		4 (3)	5 (4)	12 (6)	11 (7)	13 (9) *
Endocarditis		5 (3)	3 (2)	4 (2)	4 (3)	11 (7)
Other source ^‡^		16 (12)	22 (15)	35 (17)	13 (9)	10 (7)
Unknown source		23 (17)	10 (7)	21 (10)	28 (19)	20 (13)
**CCI**	>2	13 (10)	20 (25)	92 (49)	117 (82)	140 (94) *
≥5		7 (6)	3 (4)	33 (18)	94 (66)	112 (75) *
Not available		13	61	21	7	0
**30-day mortality**		39 (28)	41 (29)	57 (28)	57 (38)	54 (36)

^‡^ Other sources: biliary, pericardium, central nervous system. * Significant differences between the 1990–1995 and 2014–2019 periods (*p* < 0.05). CCI, Charlson’s comorbidity index; HO-HCA, hospital-onset healthcare-associated; CO-HCA, community-onset healthcare-associated; CA, community-acquired.

## Data Availability

All relevant data are available in the article or the Supplementary Materials.

## Supplementary Materials

The following supporting information can be downloaded at: https://www.mdpi.com/article/10.3390/microorganisms10122401/s1, Figure S1: Study flow diagram; Figure S2: Pangenome analysis; Table S1: Summary of MRSA bloodstream isolates included in this study; Table S2: Source of infection of MRSA-BSI episodes by acquisition; Table S3: Comparison of the clinical characteristics of the three major clones in the 2008–2019 period.
