# Supplementary material for: Molecular Epidemiology, Antimicrobial Susceptibility, and Clinical Features of Methicillin-Resistant Staphylococcus aureus Bloodstream Infections over 30 Years in Barcelona, Spain (1990–2019)
Source: Microorganisms. 2022 Dec 3;10(12):2401. doi: 10.3390/microorganisms10122401 (PMC9788191; doi:10.3390/microorganisms10122401)
Supplement: Supplementary file 1 [file microorganisms-10-02401-s001.zip › Figure S1.pdf]

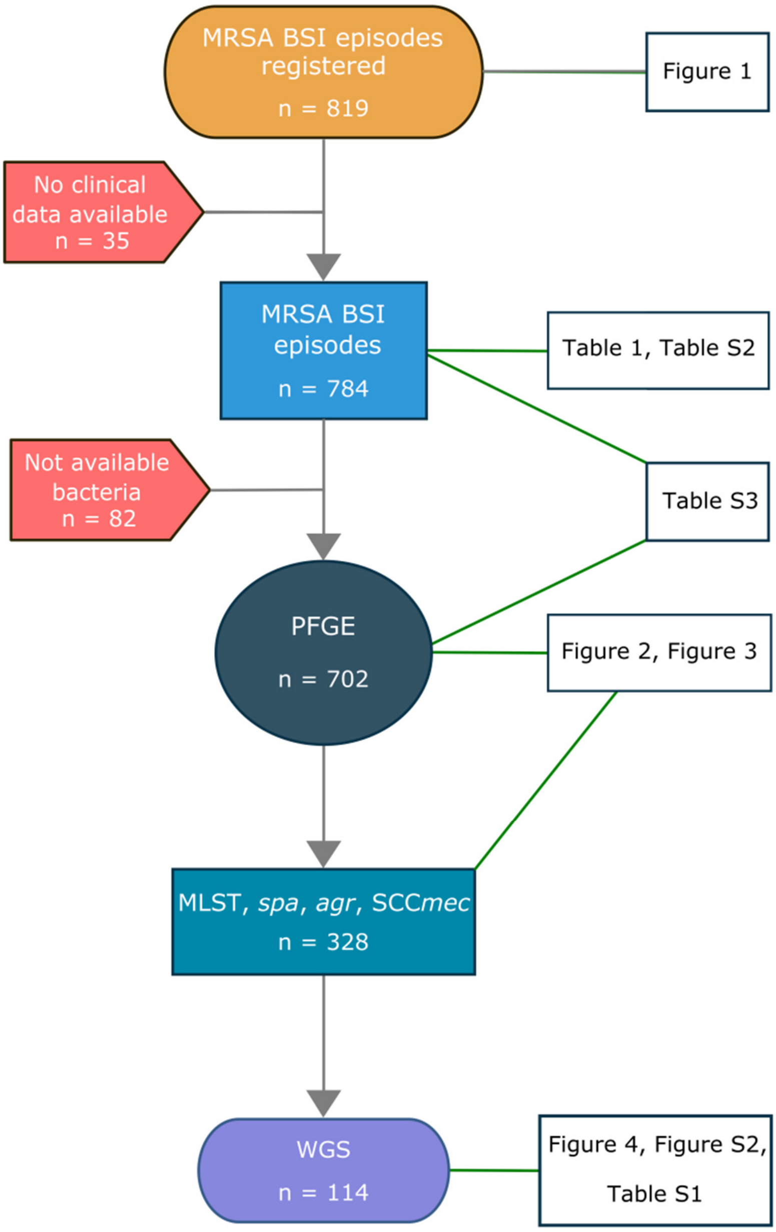

Supplementary Figure S1. **Study flow diagram.** Red polygons show the excluded episodes or isolates. The green lines connect the episodes/samples used with the respective figure/table of the work. BSI, bloodstream infection; MLST, multi locus sequence typing; SCC*mec*, staphylococcal chromosomal cassette *mec* typing; PFGE, pulsed-field gel electrophoresis; *spa*, *spa*-typing; *agr*, accessory gene regulator typing.
