# Supplementary material for: Molecular Epidemiology, Antimicrobial Susceptibility, and Clinical Features of Methicillin-Resistant Staphylococcus aureus Bloodstream Infections over 30 Years in Barcelona, Spain (1990–2019)
Source: Microorganisms. 2022 Dec 3;10(12):2401. doi: 10.3390/microorganisms10122401 (PMC9788191; doi:10.3390/microorganisms10122401)
Supplement: Supplementary file 1 [file microorganisms-10-02401-s001.zip › Figure S2.pdf]

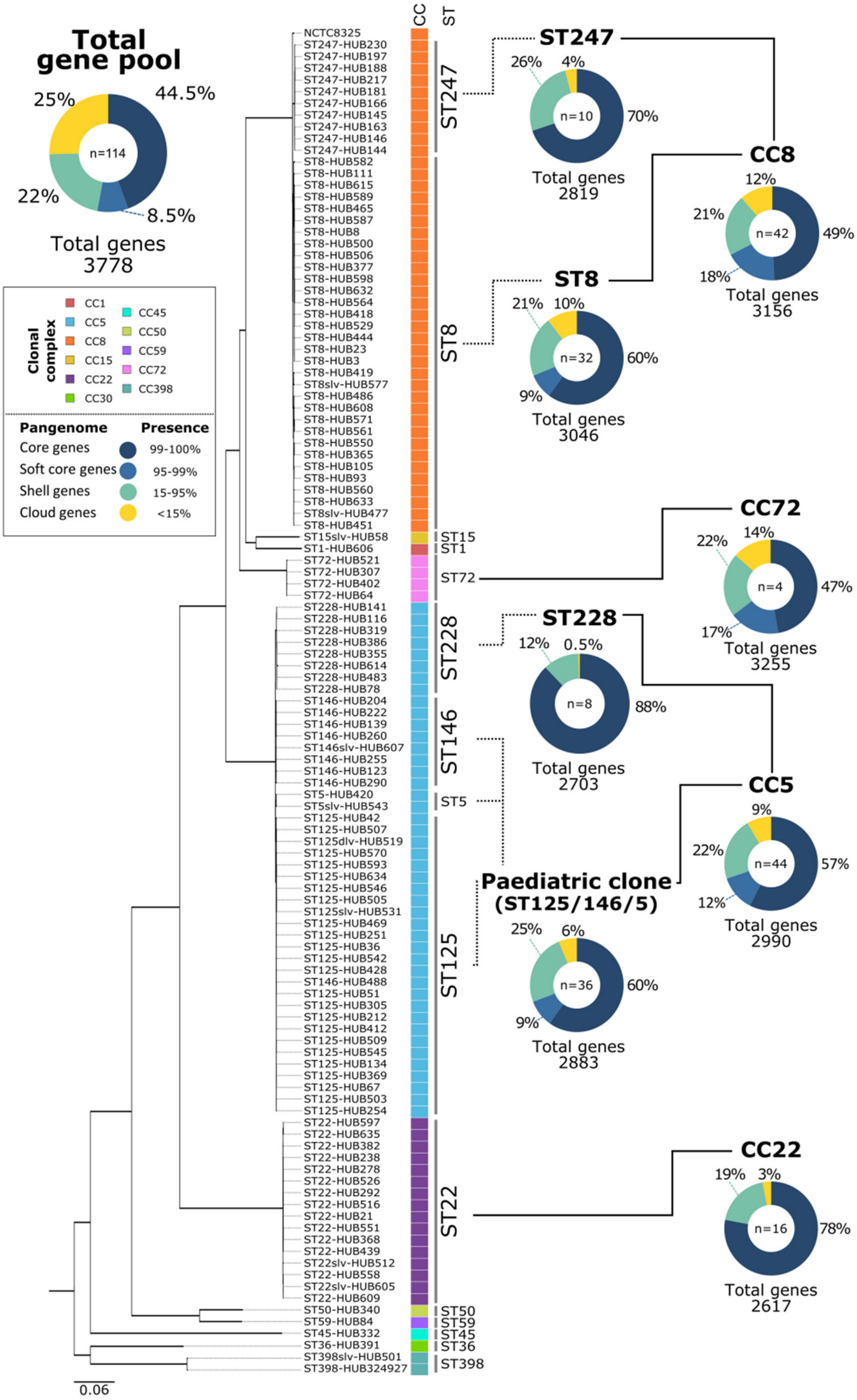

Supplementary Figure S2. Pangenome analysis. Genes were classified according to their presence in the 114 genomes analysed as: *Core* if present in ≥99% of samples, *Soft-core* between 95 and 99%, *Shell* between 15 and 95% and *Cloud* at <15%. Doughnut charts show the distribution fractions of core, shell, and cloud genes by ST, by CC and the total gene pool, with the total number of isolates of each group in the centre of the chart and the total number of genes below each chart.
