# Supplementary material for: Molecular Epidemiology, Antimicrobial Susceptibility, and Clinical Features of Methicillin-Resistant Staphylococcus aureus Bloodstream Infections over 30 Years in Barcelona, Spain (1990–2019)
Source: Microorganisms. 2022 Dec 3;10(12):2401. doi: 10.3390/microorganisms10122401 (PMC9788191; doi:10.3390/microorganisms10122401)
Supplement: Supplementary file 1 [file microorganisms-10-02401-s001.zip › Table S2.pdf]

Supplementary Table S2. Source of infection of MRSA-BSI episodes by acquisition. The table shows the total number of episodes and the percentage (in parentheses).

	**Time periods**												
	1990-1995<br>*n* = 137		1996-2001<br>*n* = 142		2002-2007<br>*n* = 207			2008-2013<br>*n* = 149			2014 – 2019<br>*n* = 149		
**Acquisition**	**HO-HCA**	**CO-HCA**	**HO-HCA**	**CO-HCA**	**HO-HCA**	**CO-HCA**	**CA**	**HO-HCA**	**CO-HCA**	**CA**	**HO-HCA**	**CO-HCA**	**CA**
**Source of infection**													
**Vascular Catheter**	74 (54)	4 (3)	65 (46)	6 (4)	33 (16)	29 (14)	-	25 (17)	9 (6)	1 (1)	11 (7)	19 (13)	-
**Endocarditis**	5 (4)	-	1 (1)	2 (1)	1 (0.5)	3 (1)	-	2 (1)	1 (1)	1 (1)	2 (1)	7 (5)	2 (1)
**Osteoarticular**	1 (1)	3 (2)	2 (1)	3 (2)	4 (2)	8 (4)	-	5 (3)	6 (4)	-	2 (1)	11 (7)	-
**Respiratory Tract**	1 (1)	-	11 (8)	3 (2)	10 (5)	7 (3)	-	5 (3)	12 (8)	-	13 (9)	9 (6)	2 (1)
**SST**	7 (5)	1 (1)	5 (4)	7 (5)	9 (4)	23 (11)	2 (1)	7 (5)	13 (9)	-	3 (2)	17 (11)	9 (6)
**Urinary**	-	2 (1)	3 (2)	2 (1)	9 (4)	13 (6)	-	5 (3)	13 (9)	3 (2)	1 (1)	9 (6)	2 (1)
**Others†**	14 (10)	-	16 (11)	2 (1)	18 (9)	9 (4)	-	8 (5)	3 (2)	1 (1)	2 (1)	6 (4)	2 (1)
**Unknown**	22 (16)	3 (2)	10 (7)	4 (3)	14 (7)	15 (7)	-	13 (9)	14 (9)	2 (1)	5 (3)	11 (7)	4 (3)

† Other sources: biliary, pericardium, central nervous system. SST, skin and soft tissue; HA, hospital acquired; HCA healthcare-associated and CA, community-acquired.
