# Supplementary material for: Molecular Epidemiology, Antimicrobial Susceptibility, and Clinical Features of Methicillin-Resistant Staphylococcus aureus Bloodstream Infections over 30 Years in Barcelona, Spain (1990–2019)
Source: Microorganisms. 2022 Dec 3;10(12):2401. doi: 10.3390/microorganisms10122401 (PMC9788191; doi:10.3390/microorganisms10122401)
Supplement: Supplementary file 1 [file microorganisms-10-02401-s001.zip › Table S3.pdf]

Supplementary Table S3. Comparison of the clinical characteristics of the three major clones in the 2008- 2019 period.

	Overall (2008-2019)						
	**Total** *n*=260	**CC5 Paediatric** *n*=112	**CC8-ST8** *n*=109	**CC22** *n*=39	***P*-value CC5 vs ST8**	***P*-value CC22 vs CC5**	***P*-value CC22 vs ST8**
**Characteristics [n (%)]**							
Age [mean±SD; (range)]	71.2±13.7 (19-98)	71.1±12.7 (31-94)	71.4±71.4 (19-98)	71.13±11.1 (39-86)	0.89	0.99	0.93
Female sex	112 (43)	51 (46)	44 (40)	17 (44)	0.49	0.854	0.851
**Source of Infection**							
Vascular Catheter	61 (23)	29 (26)	23 (21)	9 (23)	0.431	0.832	0.822
SST	45 (17)	21 (19)	18 (17)	6 (15)	0.726	0.809	-
Urinary	27 (10)	15 (13)	9 (8)	3 (8)	0.281	0.407	-
Osteoarticular	23 (9)	11 (10)	11 (10)	1 (3)	-	0.188	0.184
Respiratory Tract	37 (14)	10 (9)	19 (17)	8 (21)	0.074	0.082	0.638
Endocarditis	14 (5)	7 (6)	5 (5)	2 (5)	0.768	-	-
Other	14 (5)	6 (5)	8 (7)	-	0.591	0.339	0.111
Unknown	39 (15)	13 (12)	16 (15)	10 (26)	0.553	0.067	0.143
**Acquisition**							
CA	24 (9)	5 (4)	16 (15)	3 (8)	**0.011**	0.426	0.403
CO-HCA	142 (55)	62 (55)	60 (55)	20 (51)	-	0.711	0.711
HO-HCA	94 (36)	45 (40)	33 (30)	16 (41)	0.159	-	0.239
**CCI**							
>2	233 (91)	101 (91)	96 (90)	36 (97)	0.669	-	0.5619
≥5	137 (54)	48 (43)	60 (56)	29 (78)	0.081	**0.0008**	**0.038**
Not Available	4	1	1	2	-	0.164	0.171
**30-days Mortality**	99 (38)	41 (37)	44 (40)	14 (36)	0.583	-	0.704

‡ Other sources: biliary, pericardium, central nervous system. SST, skin and soft tissue; HO-HCA, hospital-onset healthcare-associated; CO-HCA community-onset healthcare-associated and CA, community-acquired; CCI, Charlson's comorbidity index. P-values in bold are statistically significant.
